# Effectiveness of the Combined Exercise and Cognitive Training System in Enhancing Frontal Lobe Function in Older Adults: A Randomized Control Study

**DOI:** 10.1155/bmri/4581898

**Published:** 2026-03-24

**Authors:** Kuninori Suzuki, Kazuya Saita, Michiaki Otani, Hitoshi Okamura

**Affiliations:** ^1^ Department of Psychosocial Rehabilitation, Graduate School of Biomedical and Health Sciences, Hiroshima University, Hiroshima, Japan, hiroshima-u.ac.jp; ^2^ Mazda Motor Corporation, Hiroshima, Japan, mazda.com; ^3^ Sogo Rehabilitation Laboratory, Yamaguchi, Japan

**Keywords:** cognitive function, driving behavior, dual-task, exercise, near-infrared spectroscopy

## Abstract

**Aim:**

This study was aimed at comparing the effects of a driving‐related combined exercise and cognitive training system intervention on frontal lobe function in older adults with an active control group that performed aerobic exercises.

**Methods:**

In this randomized controlled trial, 90 community‐dwelling older adults were randomly allocated (1:1) to the intervention (*n* = 45) or control groups (*n* = 45). The intervention group participated in a combined exercise‐cognitive multitask training program for six consecutive weeks, whereas the active control group performed matched‐intensity aerobic exercises during the same period. Cognitive function was assessed using the Trail Making Test Parts A and B (TMT‐A and B) and the Frontal Assessment Battery (FAB). Changes in the hemodynamics of the frontopolar cortex (FPC) during the task were measured using portable functional near‐infrared spectroscopy (fNIRS). Assessments were conducted at baseline, postintervention, and follow‐up time points.

**Results:**

The final analysis included 44 and 40 participants in the intervention and control groups, respectively. TMT‐A and B results and FAB scores showed no significant differences between the groups at any time point. Similarly, fNIRS analysis showed no significant differences in hemodynamic reactivity in the FPC between the intervention and active control groups.

**Conclusions:**

This study demonstrated that the multitask training system did not confer additional benefits beyond aerobic exercise under a low‐intensity, short‐duration protocol in older adults, with no significant improvements in cognitive performance or frontopolar hemodynamic reactivity. Future studies should incorporate extended intervention periods and evaluate outcomes in diverse demographic populations to establish the additional advantages of driving behavior‐related multitask training compared with aerobic exercises.

## 1. Introduction

Age‐related cognitive decline is a significant factor affecting the ability of older adults to drive safely [1]. In Japan, drivers aged ≥ 75 years are required to undergo cognitive testing when renewing their driver′s licenses. In the United States, physicians are legally mandated to report cognitive dysfunction in older adults that may affect driving ability [2]. These regulations reflect global efforts to ensure public road safety. Conversely, driving cessation among older adults on returning their licenses has been considered a risk factor for social isolation and progression to frailty [3, 4], raising concerns about its negative impact on health‐related quality of life (HR‐QOL). Hence, maintaining and improving cognitive function and the social environment of community‐dwelling older adults to enable driving is essential.

Driving is a complex task that requires the integration and cooperation of cognitive, motor, and somatosensory skills [5] and has been suggested to help both maintain and improve cognitive function. The prefrontal cortex, including the frontopolar cortex (FPC), is considered important because driving requires continuous cognitive and motor demands involving executive functions, particularly higher order cognitive flexibility, such as abstract set‐shifting [6]. However, the lack of a gold standard method for assessing driving aptitude [1] and the limited availability of adequate training methods tailored to driving‐related cognitive training remain important challenges. Previous driving assessment studies have reported significant differences in Trail Making Test (TMT) performance for pass/fail in on‐road driving performances [7]. The TMT Part B (TMT‐B) has frequently been selected as a brief clinical utility test for cognitive performance while driving [8–10].

Although a neuroimaging study using structural magnetic resonance imaging has reported an association between brain volume and driving performance in older drivers [11], measuring task‐related hemodynamic changes during naturalistic movements in real‐world settings is particularly desirable. Functional near‐infrared spectroscopy (fNIRS) is a noninvasive and relatively unconstrained neuroimaging technique that enables the measurement of cortical hemodynamic reactivities during dynamic cognitive tasks. A previous fNIRS study has reported an increased oxygenated hemoglobin concentration in the prefrontal cortex during driving tasks [12]. More recently, portable fNIRS systems have been developed, enabling cortical hemodynamic measurements in more naturalistic and wearable environments, and have been increasingly applied in ecological settings [13–15].

Enhancing the prefrontal cortical activity may help prevent or mitigate age‐related neural changes [16]. Systematic reviews of combined physical exercise and cognitive training have reported improvements in cognitive function in older adults, especially prefrontal lobe–related domains, such as processing speed, attention, and executive function [17, 18]. Moreover, simultaneous dual‐task training has been shown to be more effective for cognitive enhancement than sequential training approaches [19]. We previously developed an original combined exercise and cognitive training system and demonstrated its effectiveness in improving cognitive impairment in individuals with dementia [20, 21]. However, the effect of this training system on driving‐specific cognitive performance (e.g., TMT score) and prefrontal cortical hemodynamic changes in healthy older adults remains largely unexplored.

We aim to examine the effectiveness of a combined exercise and cognitive training system in improving frontal lobe–related functions in healthy older adults. Given the established cognitive benefits of aerobic exercises alone [22, 23], a rigorous evaluation of multitask training (MT) requires comparison with an active control (AC) group. Accordingly, we compared the effects of the combined training system with those of aerobic exercises in healthy older adults. If proven effective, this intervention protocol may serve as a feasible approach to support daily life, including driving, and to maintain HR‐QOL in older adults.

The hypotheses of this study were as follows:

1. Compared with aerobic exercise alone, combined MT can achieve significantly greater improvements in executive function, particularly in cognitive flexibility as measured by set‐shifting, reflected by a reduction in completion time on the TMT‐B.
2. Compared with aerobic exercise alone, combined MT can improve processing speed and frontal lobe functioning, as assessed by secondary neuropsychological tests. Additionally, combined MT would be associated with increased task‐related hemodynamic reactivity in the FPC, as measured using fNIRS.

## 2. Materials and methods

### 2.1. Study Design

This single‐center, open‐label randomized control trial was prospectively registered on the University Hospital Medical Information Network Clinical Trials Registry (UMIN‐CTR), with Registration Number UMIN000051124. The study protocol was approved by the Hiroshima University Epidemiology Ethics Review Board (IRB No. C2023‐0004). Written informed consent was obtained from all participants prior to enrollment. This study was conducted and reported in accordance with the CONSORT 2010 guideline(Table S1).

### 2.2. Participants

Community‐dwelling older adults undergoing rehabilitation at a daycare center were recruited between October 2023 and January 2024. The inclusion criteria were as follows: (1) age 65–85 years at the time of study enrollment, (2) previous experience in exercising with ergometer training, and (3) providing written consent to participate in the study. The exclusion criteria were as follows: (1) the presence of dementia diagnosed at a medical institution, (2) a history of severe cardiovascular or respiratory disease, (3) a history of motor dysfunction or pain in the upper or lower extremities, and (4) deemed unsuitable for inclusion, as decided by the principal investigator.

### 2.3. Randomization

After eligibility screening, participants were randomized (1:1) to either the MT group or the AC group. Randomization was performed using a permuted block design and a random allocation table. An independent facility staff member (R.W.) who was not involved in the study enrolled participants and assigned them to the interventions. This was an open‐label trial; neither participants nor assessors were blinded to group allocation.

### 2.4. Interventions

The intervention periods were set to 6 weeks based on previous studies [20, 21]. Participants used the combined exercise and cognitive training system to perform multiple tasks simultaneously (Figure 1a). The progression speed was determined by the participant′s ergometer pedaling speed, whereas directional control was achieved using pressure sensors mounted on the handlebar ends. The rotation angles in the clockwise and counterclockwise directions were determined based on the duration of pressure applied to the sensors on the handlebar. This configuration allowed free movement across a two‐dimensional plane on the display screen. During the task, a target trajectory was displayed (shown in yellow) on the screen in front of the participants, who were required to follow this trajectory (shown in red) by coordinating lower‐limb pedaling with finger pressure on the sensors (Figure 1b). In the MT group, participants performed multiple tasks that combined cognitive training while maintaining a constant pedaling speed of 50 rpm (Figure 1c). In the AC group, participants maintained the ergometer pedaling speed at 50 rpm without performing the tracking task. The pedaling load was set at 8–12 on the Borg Scale for both groups. Each session lasted 5 min and was conducted at least once per week. The training was discontinued if an individual did not participate in any training session for two consecutive weeks. To enhance engagement and promote continued participation, scores for both manual operation (accuracy of trajectory tracking) and pedaling performance (accuracy of tracking pedal speed) were displayed at the end of each training session.

Figure 1The combined exercise and cognitive training. (a) The combined exercise and cognitive training system (revised version), (b) The display during training in the intervention group: target trajectory (yellow lines); trajectory explored and followed by participants (red lines). (c) The display during training in the control group: A constant pedaling speed of 50 rpm is maintained.

**Figure:**
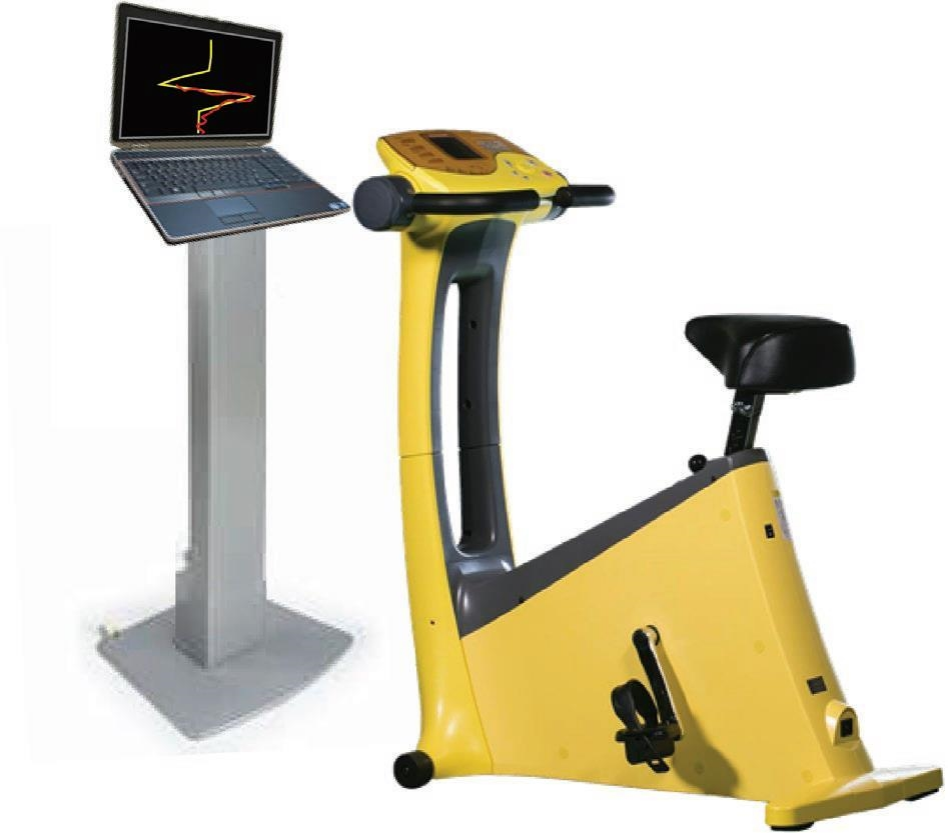
(a)

**Figure:**
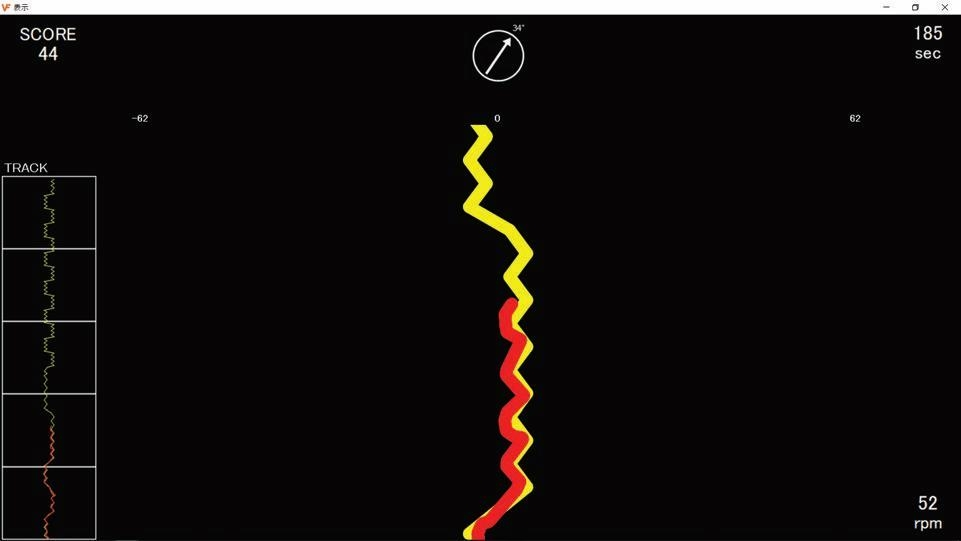
(b)

**Figure:**
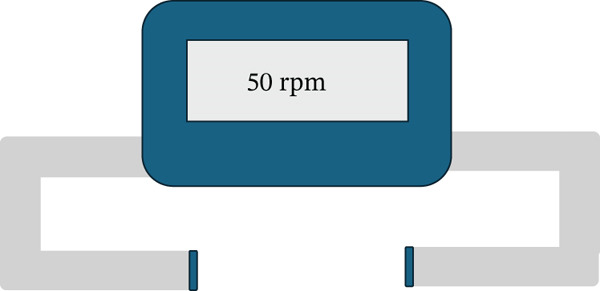
(c)

Intervention adherence was defined as the completion of the assigned training sessions. The number of sessions attended, total training time, and any adverse events were recorded for each participant. Task difficulty was programmed to increase when predefined performance thresholds were exceeded.

### 2.5. Outcome Measures

#### 2.5.1. TMT

The Japanese standardized version of the TMT [23] was used for neuropsychological tests. TMT Part A (TMT‐A) assesses processing speed, whereas TMT‐B assesses executive function, including set‐shifting ability. TMT‐A requires the sequential connection of numbers, whereas TMT‐B requires the sequential connection of numbers and Japanese hiragana characters. Shorter completion times indicate better cognitive performance. The upper limits of completion time are 180 s for TMT‐A and 300 s for TMT‐B. Individuals who exceeded the upper limit at baseline (T0) were excluded from the analysis.

#### 2.5.2. Frontal Assessment Battery

The FAB is a neuropsychological screening tool for frontal lobe function, comprising the following six subtests: conceptualization, mental flexibility, motor programming, sensitivity to interference, inhibitory control, and environmental autonomy. The total score ranges from 0 to 18 points, with higher scores indicating better performance. The Japanese version utilized in this study, which was based on the original protocol by Dubois et al. [24], demonstrated reliability and validity [25]. A cutoff score of 12 points was used for screening frontal lobe function [26].

#### 2.5.3. fNIRS Measurements

A wireless portable NIRS device (HOT‐2000; NeU Corp., Tokyo, Japan) was used. The system was operated at a sampling frequency of 10 Hz using near‐infrared light at a wavelength of 810 nm. The fNIRS device measures changes in cortical hemodynamics associated with neural activity according to the modified Beer–Lambert law [27]. This device specifically measures changes in total hemoglobin concentration (THb) and has been validated by comparison with multichannel fNIRS systems [28]. As one of the main indicators of fNIRS, THb represents the sum of oxyhemoglobin and deoxyhemoglobin concentrations. Assuming relatively stable deoxyhemoglobin levels, increases in THb concentration may reflect increased regional perfusion, corresponding to changes in oxyhemoglobin concentration. THb was employed as a biomarker of perfusion reactivity rather than a direct neural‐specific measure, acknowledging its sensitivity to systemic physiological factors, such as blood pressure and skin blood flow. The fNIRS device incorporates a multidistance optode method [29], which removes superficial scalp blood flow signals through short‐channel measurements, reducing contamination from extracerebral sources. Based on the international 10–20 system, the measurement positions were set at regions of interest (ROIs) corresponding to the bilateral FPC on the Fp1 and Fp2 lines. The FPC within the prefrontal cortex serves as an established ROI for assessing cortical hemodynamics during dual‐task performance [14, 30].

#### 2.5.4. Primary Outcome

The primary outcome was the change in TMT‐B completion time between baseline and postintervention (*Δ*TMT − B T1 − T0).

#### 2.5.5. Secondary Outcome

Secondary outcomes included TMT‐A and TMT‐B completion times and error counts, and FAB total scores at baseline (T0), postintervention (T1), and the 6‐week follow‐up (T2). Changes in THb concentration in the FPC measured by fNIRS were assessed at T0 and T1, which were the sole measurement points because the training device could only be installed during the intervention period.

#### 2.5.6. Assessment Procedure

Cognitive function was assessed at three time points: baseline (T0), postintervention (T1), and the 6‐week follow‐up (T2). Demographic characteristics, including age, sex, and current driving status, were collected through self‐reporting at baseline. At each assessment, neuropsychological tests were administered in a quiet room in the following order: TMT‐A, TMT‐B, and FAB. For the TMT, practice trials were administered before the main test, and participants unable to complete the practice trials were excluded. Following the neuropsychological assessments, participants were moved to a separate room equipped with the ergometer, where fNIRS measurements were conducted under the respective group conditions.

### 2.6. Statistical Analyses

#### 2.6.1. Sample Size Determination

The sample size was determined based on a previous study [31] that utilized the TMT‐B results as the primary outcome measures. With an expected effect size (Cohen′s *d*) of 0.627, a two‐sided significance level of 5%, and a power of 80%, the calculated total sample size was 82 (41 per group). Considering an anticipated dropout rate of 10%, the target sample size was set at 90 (45 per group).

#### 2.6.2. Baseline Characteristics

Baseline characteristics were compared between groups using the chi‐square test for categorical variables. For continuous variables, normality was assessed using the Shapiro–Wilk test, followed by an unpaired *t*‐test or Mann–Whitney *U* test, as appropriate.

#### 2.6.3. Primary Outcome Analysis

The primary outcome was the between‐group difference in TMT‐B (*Δ*TMT − B, T1 − T0), which was compared using an unpaired *t*‐test or the Mann–Whitney *U* test, as appropriate. Since *Δ*TMT‐B was defined a priori as the single primary outcome, no adjustment for multiple comparisons was applied to this analysis. Effect size for the primary outcome (*Δ*TMT‐B) was expressed as the rank‐biserial correlation (*r*), with two‐sided 95% confidence intervals (CIs).

#### 2.6.4. Secondary Outcome Analysis

Secondary neuropsychological outcomes (TMT‐A, TMT‐B, and FAB scores across time points) were analyzed using linear mixed‐effects models for repeated measures. *Group* (MT vs. AC) and *Time* (T0, T1, and T2) were included as fixed effects, and individuals were included as a random effect. Repeated measurements within individuals were modeled using a compound symmetry covariance structure, and parameters were estimated using restricted maximum likelihood methods. Mixed‐effects models were adjusted for age, sex, driving status, and baseline outcome values of each outcome, as appropriate. Effect sizes for secondary outcomes were reported as *η* *p* ^2^ with 95% CI and adjusted fixed‐effect estimates (*β*) with two‐sided 95% CIs. In multiple comparisons for secondary outcomes, *p* values were controlled using the false discovery rate (FDR).

To contextualize the clinical relevance of observed changes, TMT completion times were compared with age‐stratified normative data for the Japanese population [23], and the results were expressed as *Z*‐scores. FAB scores were interpreted against the established clinical cutoff of 12 points for frontal lobe dysfunction [26]. Established MCID thresholds for TMT were not applied because available estimates were derived from substantially longer follow‐up periods (19–32 months) compared with the 6‐week intervention in this study and population‐specific MCID values for Japanese cohorts have not been established.

#### 2.6.5. fNIRS Preprocessing and Analysis

The wearable fNIRS preprocessing pipeline followed established principles from published methodology [32–35], adapted to the portable continuous wave fNIRS device used in this study. Waveform data with notable motion artifacts detected as an error signal by the fNIRS device were excluded from the analysis. In addition, signals from short‐separation channels were used to regress out superficial physiological components, enabling the separation of cortical hemodynamic reactivities from skin blood flow–related changes [28]. A bandpass filter (threshold: 0.0001–0.05 Hz) was used for the filtering process. The cutoff frequency was set lower than in previous postural and walking tasks [34] to capture slow upward or downward trends over the 5‐min measurement. Because such trends correspond to a quarter‐period in 5 min of sin waveform, a full‐period sin waveform would take 20 min. The cutoff frequency was therefore set based on the reciprocal of 20 min, enabling extraction of these slow trends. The smoothed average waveforms were baseline‐corrected with the dual‐task onset set at zero. To enable comparisons across channels and individuals, the changes in THb concentration were converted to *Z*‐scores. This normalization was achieved by dividing the averaged waveforms by the standard deviation of THb concentration during the 30‐s control task period. To account for the ascending trend component owing to prolonged measurements, the fNIRS data analysis interval ranged from 30 s before starting the dual task (during the control task) to 120 s after starting the task.

Normalized THb data were analyzed using a linear mixed‐effects model for repeated measures, with *Group* (MT vs. AC) as a between‐subject factor and *Time* (T0 vs. T1) and *Hemisphere* (left FPC vs. right FPC) as within‐subject factors. Individuals were modeled as a random intercept. The primary fNIRS endpoint was prespecified as the *Group × Time* interaction, reflecting intervention‐specific changes in FPC hemodynamic reactivity. The omnibus model tested main effects of *Group*, *Time*, and *Hemisphere*, as well as all two‐way interactions (*Group × Time*, *Group × Hemisphere*, and *Time × Hemisphere*) and the three‐way interaction (*Group × Time × Hemisphere*). Post hoc analyses were performed using pairwise comparisons with FDR correction. Effect sizes are reported as fixed‐effect estimates (*β*) with 95% CIs. Since THb was *z*‐score normalized within individuals, *β* coefficients can be interpreted as standardized effect sizes in within‐subject standard deviation units.

The fNIRS data were preprocessed using Python 3.12 (Python Software Foundation, Wilmington, Delaware, United States). All statistical analyses were performed using JMP Pro 18.1.0 (SAS Institute, Inc., Cary, North Carolina, United States) with a two‐tailed significance level of *p* < 0.05.

## 3. Results

**Figure 2:**
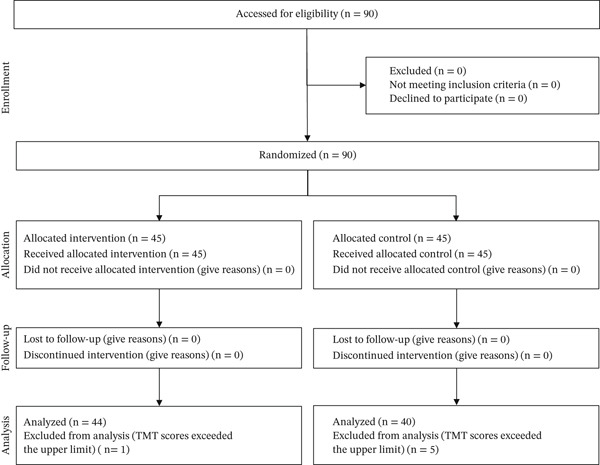
Study flow chart. TMT, Trail Making Test.

A total of 90 individuals were enrolled, and 45 were randomly allocated to each (intervention and control) group. At T0, six participants whose TMT results exceeded the upper limit were excluded from the analysis. No dropouts due to exercise intolerance occurred in either group. The follow‐up period lasted until April 2024, and the study was completed as planned. Finally, the data of 44 and 40 participants in the intervention and control groups, respectively, were included in the analysis (Figure 2). Between‐group comparisons of the T0 assessment revealed statistically significant differences in TMT‐A results but not in other characteristics or cognitive assessments (Table 1).

**Table 1:** Comparison of baseline characteristics and clinical outcomes between groups.

	Total	MT group	AC group	
	*N* = 84median [IQR]	*N* = 44median [IQR]	*N* = 40median [IQR]	*p*
**Age**, years	79 [75–82.3]	79 [75–82.3]	80 [74.8–82.3]	0.65^a^
**Sex**, female (%)	52 (61.9)	27 (61.4)	25 (61.5)	0.91^b^
**Driving**, no (%)	64 (76.2)	35 (79.6)	29 (72.5)	0.45^b^
**TMT-A**, seconds	63 [52–82.3]	58.4 [48.0–70.5]	67.3 [53.8–94.5]	0.02^a^
**TMT-A**, errors	0 [0–0]	0 [0–0]	0 [0–0]	0.14^a^
**TMT-B**, seconds	119.5 [98.2–156]	115.5 [97.5–156]	120.5 [98.8–158.4]	0.94^a^
**TMT-B**, errors	1 [0–2]	1 [0–1.8]	1 [0–2]	0.74^a^
**FAB**, points	14 [13–16]	15 [13–16]	14 [13–15.3]	0.49^a^

*Note:* Statistically significant, *p* < 0.05.

Abbreviations: AC, active control; FAB, Frontal Assessment Battery; TMT‐A, Trail Making Test Part A; TMT‐B, Trail Making Test Part B; MT, multitask training.

^a^Mann–Whitney *U* test.

^b^Chi‐square test.

**Figure 3:**
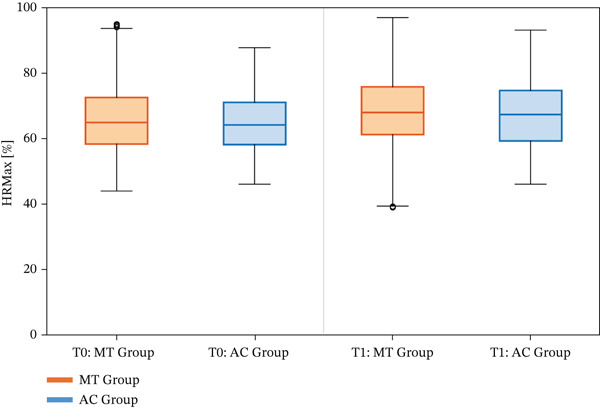
Between‐group comparisons of maximum heart rate (%HRMax) at baseline (T0) and postintervention (T1). Data are presented as median [IQR]. MT, multitask training; AC, active control.

Intervention adherence and dosing were as follows: participants in the intervention group completed a mean of 7.3 ± 2.9 sessions (range: 4.0–18.0), corresponding to a total of 36.5 ± 14.7 min (range: 20.0–90.0), whereas participants in the control group completed a mean of 8.4 ± 3.3 sessions (range: 3.0–18.0), corresponding to a total of 41.8 ± 16.7 min (range: 15.0–90.0). All participants completed the assigned sessions, and no adverse events related to training occurred. Task difficulty was prespecified to progress when performance exceeded the predefined threshold; however, no participants met the progression criteria during the study period. Despite slight individual variations, the exercise load of the individuals′ heart rates ranged from approximately 70 to 120 beats per minute for both the intervention and AC groups (Figure 3).

### 3.1. Neuropsychological Outcome

No statistically significant between‐group difference was observed in the primary outcome *Δ*TMT − B (T1 − T0) (median [interquartile range]: MT group, 5.5 [−14.5, 38.13] vs. AC group, 5.5 [−10, 33.5]; *U* = 866, *p* = 0.893, rank‐biserial *r* = −0.016, 95% CI [−0.265, 0.233]).

Regarding the secondary outcomes assessed using the adjusted mixed model for repeated measures, TMT‐A completion times (adjusted for age, sex, driving status, and baseline TMT‐A) showed no significant *Group × Time* interaction (*F*_(2, 164)_ = 1.91, *p* = 0.151, *η* *p* ^2^[95*%*CI] = 0.023 [0.000, 0.007]), indicating that temporal changes did not differ between groups, and no significant main effect was observed for *Group* (*F*_(1, 237.3)_ = 2.10, *p* = 0.148, *η* *p* ^2^[95*%*CI] = 0.009 [0.000, 0.032]). A significant main effect of *Time* was observed (*F*_(2, 164)_ = 23.51, *p* < 0.001, *η* *p* ^2^[95*%*CI] = 0.223 [0.113, 0.333]). Post hoc analysis showed shorter completion times at T1 than at T0 (*β* = −17.30 s, 95% CI [−22.94, −11.66], *p* < 0.001) and longer completion times at T2 than at T1 (*β* = 7.44 s, 95% CI [1.80, 13.08], *p* = 0.010).

For TMT‐B completion times (adjusted for age, sex, and driving status), neither a significant *Group × Time* interaction was observed (*F*_(2, 164)_ = 0.08, *p* = 0.927, *η* *p* ^2^[95*%*CI] = 0.001 [0.000, 0.010]), nor was there a significant main effect of *Group* (*F*_(1, 128.6)_ = 0.15, *p* = 0.695, *η* *p* ^2^[95*%*CI] = 0.001 [0.000, 0.013]). A significant main effect of *Time* was detected (*F*_(2, 164)_ = 11.00, *p* < 0.001, *η* *p* ^2^ [95*%*CI] = 0.118 [0.027, 0.209]). Post hoc analysis indicated no significant difference between T0 and T1 (*β* = 14.29 s, 95% CI [−0.28, 28.85], *p* = 0.055), whereas TMT‐B completion times were significantly shorter at T2 than at T1 (*β* = −21.56 s, 95% CI [−36.12, −7.00], *p* = 0.004).

For FAB score (adjusted for age, sex, and driving status), neither a significant *Group × Time* interaction (*F*_(2, 164)_ = 0.53, *p* = 0.592, *η* *p* ^2^ [95*%*CI] = 0.006 [0.000, 0.030]) nor a significant main effect of *Group* (*F*_(1, 175.2)_ = 0.78, *p* = 0.377, *η* *p* ^2^ [95*%*CI] = 0.004 [0.000, 0.024]) was observed. A significant main effect of *Time* was observed (*F*_(2, 164)_ = 14.98, *p* < 0.0001, *η* *p* ^2^ [95*%*CI] = 0.154 [0.055, 0.254]). FAB scores improved significantly at T1 compared with those at T0 (*β* = 0.85 points, 95% CI [0.21, 1.49], *p* = 0.009), whereas the change from T1 to T2 did not reach significance (*β* = 0.58 points, 95% CI [−0.06, 1.21], *p* = 0.076).

Regarding clinical relevance, both groups showed improvements in the proportion of participants performing within normal limits. For TMT‐A, the proportion within 2 SD of normative values increased from T0 to T1 in both groups. For FAB, the proportion scoring above the clinical cutoff of 12 points also increased in both groups. The detailed changes in the values are presented in Tables 2 and 3.

**Table 2:** Comparison of cognitive outcomes between groups across time points.

Outcome		Group	Baseline (T0)	After intervention (T1)	Follow‐up (T2)
**TMT-A**	Seconds	MT	62.64 [55.61, 69.66]	52.90 [47.04, 58.77]	57.86 [52.88, 62.85]
		AC	74.89 [66.58, 83.20]	57.59 [50.87, 64.31]	65.03 [58.22, 71.83]
	*z*‐score	MT	1.50 [0.78, 2.23]	0.50 [‐0.09, 1.09]	0.96 [0.47, 1.45]
		AC	2.61 [1.76, 3.45]	0.84 [0.16, 1.53]	1.61 [0.92, 2.30]
	*z*‐score ≤ 2SD, *n* (%)	MT	23 (52.27%)	30 (68.18%)	27 (61.36%)
		AC	13 (32.50%)	24 (60.00%)	18 (45.00%)
**TMT-B**	Seconds	MT	129.57 [116.28, 142.86]	145.88 [125.33, 166.42]	120.34 [103.98, 136.71]
		AC	133.47 [116.84, 150.11]	147.76 [126.24, 169.27]	126.2 [108.57, 143.83]
	*z*‐score	MT	1.45 [0.92, 1.98]	2.12 [1.28, 2.95]	1.05 [0.38, 1.73]
		AC	1.50 [0.81, 2.19]	1.95 [1.08, 2.81]	1.24 [0.48, 2.01]
	*z*‐score ≤ 2SD, *n* (%)	MT	25 (56.82%)	22 (50.00%)	26 (59.09%)
		AC	23 (57.50%)	16 (41.03%)	22 (55.00%)
**FAB**	FAB, points	MT	14.45 [13.91, 15.0]	15.16 [14.64, 15.68]	15.43 [14.89, 15.97]
		AC	14.1 [13.39, 14.81]	14.95 [14.25, 15.65]	15.53 [14.89, 16.16]
	> 12 points, *n* (%)	MT	38 (86.36%)	43 (97.73%)	43 (97.73%)
		AC	33 (82.50%)	34 (85.00%)	37 (92.50%)

*Note:* Values are mean (95% confidence interval [CI]).

Abbreviations: AC, active control group; FAB, Frontal Assessment Battery; MT, multitask training group; TMT‐A, Trail Making Test Part A; TMT‐B, Trail Making Test Part B.

**Table 3:** Mixed‐effects model for repeated measure results for neuropsychological outcomes.

Outcome	Effect	F (df)	*p*	*η* *p* ^2^[95% CI]	*β* [95% CI]
**TMT-A** ^**†**^	*Group × time*	1.91 (2, 164)	0.151	0.023 [0.000, 0.007]	—
	*Group*	2.10 (1, 237.3)	0.148	0.009 [0.000, 0.032]	−4.23 [−9.96, 1.51]
	*Time*	23.51 (2, 164)	< 0.001	0.223 [0.113, 0.333]	—
	[T1–T0]	—	< 0.001	—	−17.30 [−22.94, −11.66]
	[T2–T1]	—	0.010	—	7.44 [1.80, 13.08]
**TMT-B** ^**‡**^	*Group × time*	0.08 (2, 164)	0.927	0.001 [0.000, 0.010]	—
	*Group*	0.15 (1, 128.6)	0.695	0.001 [0.000, 0.013]	−4.79 [−28.91, 19.32]
	*Time*	11.00 (2, 164)	< 0.001	0.118 [0.027, 0.209]	—
	[T1–T0]	—	0.055	—	14.29 [−0.28, 28.85]
	[T2–T1]	—	0.004	—	−21.56 [−36.12, −7.00]
**FAB** ^**‡**^	*Group × time*	0.53 (2, 164)	0.592	0.006 [0.000, 0.030]	—
	*Group*	0.78 (1, 175.2)	0.377	0.004 [0.000, 0.024]	0.37 [−0.45, 1.19]
	*Time*	14.98 (2, 164)	< 0.001	0.154 [0.055, 0.254]	—
	[T1–T0]	—	0.009	—	0.85 [0.21, 1.49]
	[T2–T1]	—	0.076	—	0.58 [−0.06, 1.21]

*Note:* Dagger denotes adjusted for age, sex, driving status, and baseline TMT‐A. Double dagger denotes adjusted for age, sex, and driving status.

Abbreviations: *β*, fixed‐effect estimate; CI, confidence interval; df, degrees of freedom; FAB, Frontal Assessment Battery; pts, points; s, seconds; TMT‐A, Trail Making Test Part A; TMT‐B, Trail Making Test Part B; T0, baseline assessment; T1, after intervention assessment; T2, follow‐up assessment.

### 3.2. fNIRS Results

**Figure 4:**
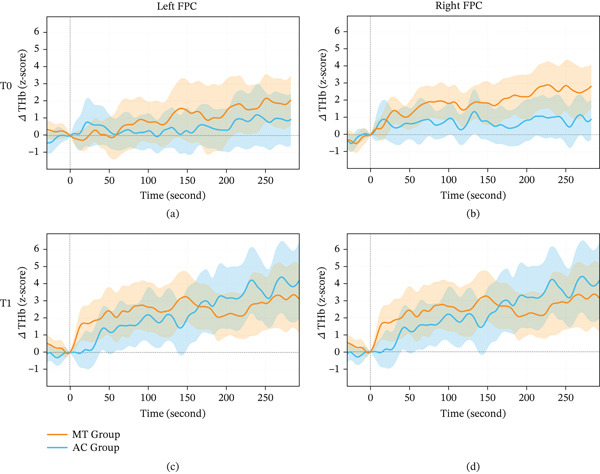
Between‐group comparisons of mean normalized waveforms showing changes in hemoglobin concentration at baseline (T0) and postintervention (T1). (a) Changes in the left frontopolar cortex (FPC) at T0. (b) Changes in the right FPC at T0. (c) Changes in the left FPC at T1. (d) Changes in the right FPC at T1. The orange line represents the multitasking (MT) group; the blue line represents the active control (AC) group. Shaded ribbons indicate 95% confidence intervals. *Δ*THb [*z*‐score], *Z*‐score normalized change in total hemoglobin concentration.

For the three‐way mixed model for repeated measures, no significant three‐way interaction (*Group × Time × Hemisphere*) was observed (*F*_(1, 146.3)_ = 0.995, *p* = 0.320; *β* = 0.36, 95% CI [−0.35, 1.06]). Further, no significant two‐way interactions were detected for *Group × Time* (primary fNIRS endpoint; *F*_(1, 146.3)_ = 0.105, *p* = 0.747; *β* = −0.12, 95% CI [−0.82, 0.59]), *Group × Hemisphere* (*F*_(1, 258.8)_ = 0.292, *p* = 0.589; *β* = −0.15, 95% CI [−0.72, 0.41]), or *Time × Hemisphere* (*F*_(1, 146.3)_ = 0.786, *p* = 0.377; *β* = −0.32, 95% CI [−1.12, 0.39]). Regarding main effects, a significant effect of *Time* was observed (*F*_(1, 146.3)_ = 4.86, *p* = 0.029; *β* = 0.78, 95% CI [0.08, 1.49]), indicating increased THb concentration from T0 to T1 across both groups and hemispheres. No significant main effects were found for *Group* (*F*_(1, 258.5)_ = 2.19, *p* = 0.140; *β* = 0.42, 95% CI [−0.14, 0.98]) or *Hemisphere* (*F*_(1, 285.8)_ = 0.83, *p* = 0.363; *β* = −0.26, 95% CI [−0.82, 0.30]). The detailed changes in the values are presented in Table 4, and mean normalized THb waveforms at T0 and T1 for both hemispheres are shown in Figure 4.

**Table 4:** Mixed‐model analyses of fNIRS changes in THb.

Effect	F (df)	*p*	*β* [95% CI]
**Three-way interaction**			
*Group × time × hemisphere*	0.995 (1, 146.3)	0.320	0.36 [−0.35, 1.06]
**Two-way interaction**			
*Group × time*	0.105 (1, 146.3)	0.747	−0.12 [−0.82, 0.59]
*Group × hemisphere*	0.292 (1, 285.8)	0.589	−0.15 [−0.72, 0.41]
*Time × hemisphere*	0.786 (1, 146.3)	0.377	−0.32 [−0.12, 0.39]
**Main effect**			
*Group*	2.187 (1, 258.8)	0.140	0.42 [−0.14, 0.98]
*Time*	4.860 (1, 146.3)	0.029	0.78 [0.08, 1.49]
*Hemisphere*	0.832 (1, 285.8)	0.363	−0.26 [−0.82, 0.30]

*Note:* THb was *z*‐score normalized within participant; *β* coefficients represent standardized effect sizes in within‐subject SD units. The *Group × Time* interaction was prespecified as the primary fNIRS endpoint, *p* < 0.05.

Abbreviations: *β*, fixed‐effect estimate; CI, confidence interval; df, degrees of freedom; THb, total hemoglobin concentration.

## 4. Discussion

In this study, we examined the effect of a revised combined exercise and cognitive training system on frontal lobe functions compared with those of matched‐intensity aerobic exercises in community‐dwelling older adults. Contrary to our hypothesis, no significant between‐group differences were observed in the primary outcome (*Δ*TMT‐B) or in secondary neuropsychological outcomes (TMT‐A and FAB). Confirmatory fNIRS analysis revealed only a significant main effect of *Time*, with THb concentration increasing from T0 to T1 across both groups and hemispheres, and no significant interactions. These findings indicate that, under the conditions employed in the present study, the multitask intervention did not confer behavioral or neurophysiological benefits beyond those associated with aerobic exercise alone.

The absence of significant between‐group differences contrasts with a previous study [31], which reported significant improvements in TMT‐B following dual‐task training. Changes in the secondary outcomes—TMT‐A and FAB scores—in the MT group were also not significantly different from those in the AC group. However, within‐group comparisons revealed significant improvements at T1 in both groups, with FAB scores maintained at T2. These within‐group improvements are consistent with previous studies using our original device [20, 36], although no additional benefits of dual tasks were detected under present study conditions.

One likely explanation is the use of an AC condition with matched exercise frequency and intensity in the present study. Aerobic exercise has well‐established beneficial effects on cognitive performance in older adults [37, 38]. Although our rigorous design minimized bias, it might have made it difficult to detect any additional benefits of the dual‐task component beyond the effects of aerobic exercise alone. A systematic review of dual‐task training has emphasized the importance of incorporating AC groups and comprehensively reporting outcomes to mitigate publication bias [39]. Our dual‐task protocol demonstrated improvements comparable with but not superior to those achieved through aerobic exercise alone within the present sample and dose condition. These findings provide valuable insights for refining research protocols in future dual‐task or MT studies.

The overall increase in THb from T0 to T1 observed across both groups and hemispheres is consistent with a general time‐related change not specific to the intervention, possibly reflecting the effects of nonspecific factors such as task familiarization, changes in physiological arousal, or broader adaptations associated with repeated testing or regular physical activity. The small effect sizes and wide CIs further suggest that any intervention‐specific modulation of frontopolar hemodynamics, if present, was likely modest under the utilized protocol conditions.

Another important consideration is the intentionally low‐intensity, short‐duration nature of the intervention protocol. This conservative approach was designed to prioritize feasibility and minimize participant burden in an older population, potentially limiting the magnitude of both behavioral and neurophysiological effects. Furthermore, only 23.8% of participants were active drivers, suggesting a limited overlap with populations most likely to benefit from driving‐related multitasking interventions. In addition, the relatively high mean age of the sample (79 years) compared with those in previous studies [20, 21] might have influenced the sensitivity of responsiveness to cognitive training. Consistent with this interpretation, little individualized adjustment of task difficulty was implemented, which might have limited engagement with the intended dual‐task challenge. Future studies targeting younger old adults (e.g., aged 60–70 years) or functionally active individuals who regularly engage in complex real‐world multitasking, such as active drivers, may be better positioned to detect intervention‐specific effects.

This study had some limitations. First, its generalizability was restricted owing to the single‐center study design, the relatively high mean age (79 years) of the sample, and the large proportion of nondriver (76.2%) community‐dwelling older adults. Second, fNIRS measurements were restricted to the FPC, excluding other important cortical regions, such as those related to the connectivity of the prefrontal–parietal network known to support executive control. Third, no longitudinal fNIRS follow‐up was conducted, limiting inferences regarding the persistence of the observed hemodynamic changes. Additionally, THb was employed as an index of perfusion reactivity rather than a direct neural‐specific measure. Although the device incorporates a multidistance optode method to reduce contamination from superficial scalp blood flow, residual influences from extracerebral sources cannot be definitively excluded. Therefore, THb changes should be interpreted conservatively as indicators of hemodynamic reactivity rather than direct neural activation. Future research should focus on dose escalation, including increased session duration, higher training frequency, and extended total intervention length, and incorporate a longer follow‐up to assess the durability of the effects. Additionally, broader cortical coverage in neuroimaging assessments would help clarify the neural mechanisms underlying dual‐task training.

## 5. Conclusion

This study demonstrated that a low‐intensity, short‐duration dual‐task intervention did not confer additional benefits in executive function or frontopolar hemodynamic reactivities beyond those achieved with aerobic exercise alone. These findings provide important evidence for refining future dual‐task training protocols, underscoring the need for higher training doses, longer intervention periods, broader cortical coverage, and careful selection of target populations. Such refinements will be essential to determine whether multitasking–based interventions can elicit robust and sustained cognitive and neurophysiological benefits in older adults.

## Author Contributions

**Concept and design:** Kuninori Suzuki, Kazuya Saita, Michiaki Otani, and Hitoshi Okamura; **acquisition of subjects and/or data:** Michiaki Otani; **analysis and interpretation of data:** Kazuya Saita and Hitoshi Okamura; **preparation of the manuscript**: Kuninori Suzuki, Kazuya Saita, and Hitoshi Okamura.

## Funding

This study was supported in part by Mazda Motor Corporation and the Grants‐in‐Aid for Scientific Research (KAKENHI) from the Japan Society of the Promotion of Science (Grant No.: JP23K14734).

## Ethics Statement

This study was approved by the Ethical Review Board of the Epidemiological Research of Hiroshima University (C2023‐0004).

## Consent

Written informed consent was obtained from all participants.

## Conflicts of Interest

The research instrument was funded by Mazda Corporation. The first author (Kuninori Suzuki), an employee of the company, did not conduct any experiments or data analyses. This author′s conflicts of interest were disclosed to and approved by the Conflict‐of‐Interest Committee prior to the start of the study. The other co‐authors declared no conflicts of interest.

## Supporting information


**Supporting Information** Additional supporting information can be found online in the Supporting Information section. Table S1: CONSORT 2010 checklist of information to include when reporting a randomized control trial.

## Data Availability

The data that support the findings of this study are available from the corresponding author upon reasonable request.
